# Editorial: The Role of Platelet Activation in the Pathophysiology of HIV, Tuberculosis and Pneumococcal Disease

**DOI:** 10.3389/fimmu.2021.737016

**Published:** 2021-07-30

**Authors:** Theresa M. Rossouw, Charles Feldman

**Affiliations:** ^1^Department of Immunology, Faculty of Health Sciences, University of Pretoria, Pretoria, South Africa; ^2^Department of Internal Medicine, Faculty of Health Sciences, University of the Witwatersrand, Johannesburg, South Africa

**Keywords:** platelet activation, HIV, tuberculosis, pneumococcus (*Streptococcus pneumoniae*), platelet aggregation

Platelets, best known as essential effector cells in coagulation and hemostasis, are increasingly recognized as major inflammatory cells that play a role in the innate and adaptive arms of the immune system. The interaction of platelets with various cell types of the innate immune system, particularly neutrophils, as well as with structural cells of the vascular endothelium, induces the release of platelet-derived mediators, thus exerting a protective effect in the physiologic response to diseases and control of invading microorganisms (1). However, it is also known that contrary to this protective anti-infective role of a tightly controlled activation of platelets, aberrant platelet activation can lead to systemic inflammation, with organ dysfunction and thrombotic complications in both acute and chronic inflammatory disorders of infective origin. It is, therefore, pleasing that this Research Topic of Frontiers in Immunology contains a series of up-to-date articles on the role of platelet activation in the pathophysiology of three important host infections, namely the human immunodeficiency virus (HIV), *Mycobacterium tuberculosis* (TB) and *Streptococcus pneumoniae* (pneumococcus).

The article entitled “Platelets and their role in the pathogenesis of cardiovascular events in patients with community-acquired pneumonia” by Feldman and Anderson is a review article, concentrating primarily on the role of platelets in the host defense against infection, particularly pneumococcal infection, and describes the current evidence in the literature for how platelet activation may contribute to cardiovascular complications in patients with community-acquired pneumonia. This includes a review of work from the authors’ own laboratory, which uniquely showed that the pneumococcal toxin, pneumolysin, considered by many to be the most important virulence factor of the pneumococcus bacterium, activated platelets, activated formation of neutrophil extracellular traps, and mediated heterotypic aggregation of neutrophils and platelets *in vitro*. It is thought that the cytolytic action of pneumolysin itself, together with its activation of platelets, plays a major role in the occurrence of cardiac complications in pneumococcal pneumonia.

The article entitled “Platelet activation and the immune response to tuberculosis” by Kirwan et al. is a review article that describes the emerging evidence that platelets play a significant role in TB immunopathology. Patients with TB disease often have thrombocytosis, which correlates with disease severity (clinically and radiologically advanced disease) and is associated with a hypercoagulable profile. It is thought that platelets drive the pathology of TB through their effects on other immune cells, especially monocytes, leading to upregulation of activation, increased matrix metalloproteinase secretion, and enhanced phagocytosis. Patients with TB exhibit changes in platelet structure and function. Similar to what was described for pneumococcal infections, activated platelets interact with leukocytes, which facilitates the recruitment of these cells to the site of infection, and is associated with the formation of platelet-monocyte and platelet-neutrophil aggregates and the release of neutrophil extracellular traps, the latter being associated with severe TB-associated lung damage and long-term sequelae.

Interestingly, both these manuscripts discuss the emerging concept, although still largely unexplored, of the use of platelet-targeted interventions in both conditions. For community-acquired pneumonia, these include pharmacological strategies that directly target platelet activation and their mediators of thrombosis and inflammation, as well as additional indirect adjunctive therapies, such as macrolide antibiotic therapy, that largely target pneumolysin-induced platelet activation. For TB, these have been repurposed anti-platelet and anti-inflammatory non-steroidal anti-inflammatory drugs. The authors of both these manuscripts are to be commended for their cutting-edge contributions to this Research Topic of the Journal.

Further to exploring the role of platelets in the pathophysiology of TB disease, the article entitled “Bedaquiline suppresses ADP-mediated activation of human platelets *in vitro* via interference with phosphatidylinositol 3-kinase” by Tintinger et al. shows the results of *in-vitro* experiments that examined the impact of bedaquiline, an anti-TB agent used for the treatment of multidrug-resistant TB, on human platelets. The authors demonstrated dose-related reduction of calcium mobilization, CD62P expression and aggregation of ADP-activated platelets, seemingly via inhibition of phosphatidylinositol 3-kinase-mediated phosphorylation of the serine/ threonine kinase, Akt1, an important facilitator of alpha granule release from platelets. They speculate that this anti-platelet effect could be secondary to the cationic amphiphilic properties of this agent and could play a role in ameliorating the risk of TB-associated cardiovascular disease. Further investigation of this finding in the clinical setting is clearly needed.

The third pathogen of interest, HIV, is discussed in two reviews and one research article. The latter authored by Steel et al. entitled “Differential responsiveness of the platelet biomarkers, systemic CD40 ligand, CD62P, and platelet-derived growth factor-BB, to virally-suppressive antiretroviral therapy” compares three circulating biomarkers of platelet activation between treatment-naive and virally suppressed participants. The authors found that sCD40L and sCD62P were significantly elevated in the treatment-naive group and identified sCD40L, in particular, as a potential biomarker of successful HIV treatment. If confirmed in the clinical setting, this biomarker could augment current monitoring strategies.

Madzime et al. reviewed the “Interactions of HIV and antiretroviral therapy with neutrophils and platelets”. The authors describe the role of platelets in harboring and disseminating HIV, as well as the contribution of persistent platelet activation and dysfunction in the development of systemic immune activation and an inflamed vasculature, both of which could increase the risk for cardiovascular disease. They highlight the fact that certain antiretroviral medications, most notably abacavir, as well as ritonavir-boosted lopinavir and darunavir, have all been linked to an increased risk of CVD.

The final article in this Research Topic by E Pretorius is a review entitled “Platelets in HIV: A guardian of host defense or transient reservoir of the virus?”. Apart from detailing the inhibitory action of platelets with regards to HIV, this review delves deeper into the findings of recent studies that demonstrated that the virus can temporarily hide from the immune system inside platelets. Such platelet reservoirs may be of particular significance during treatment interruption. Finally, the risk of platelet hypercoagulation secondary to platelet complex formation with immune cells and erythrocytes, is highlighted, a theme that has been recurrent throughout this Research Topic.

**Figure 1** summarizes the role of platelet activation in the pathophysiology of HIV, TB and pneumococcal disease Researchers in these fields should consider exploring directed monitoring and treatment strategies, especially the possibilities of platelet-targeted therapies to improve treatment outcomes.

**Figure 1:**
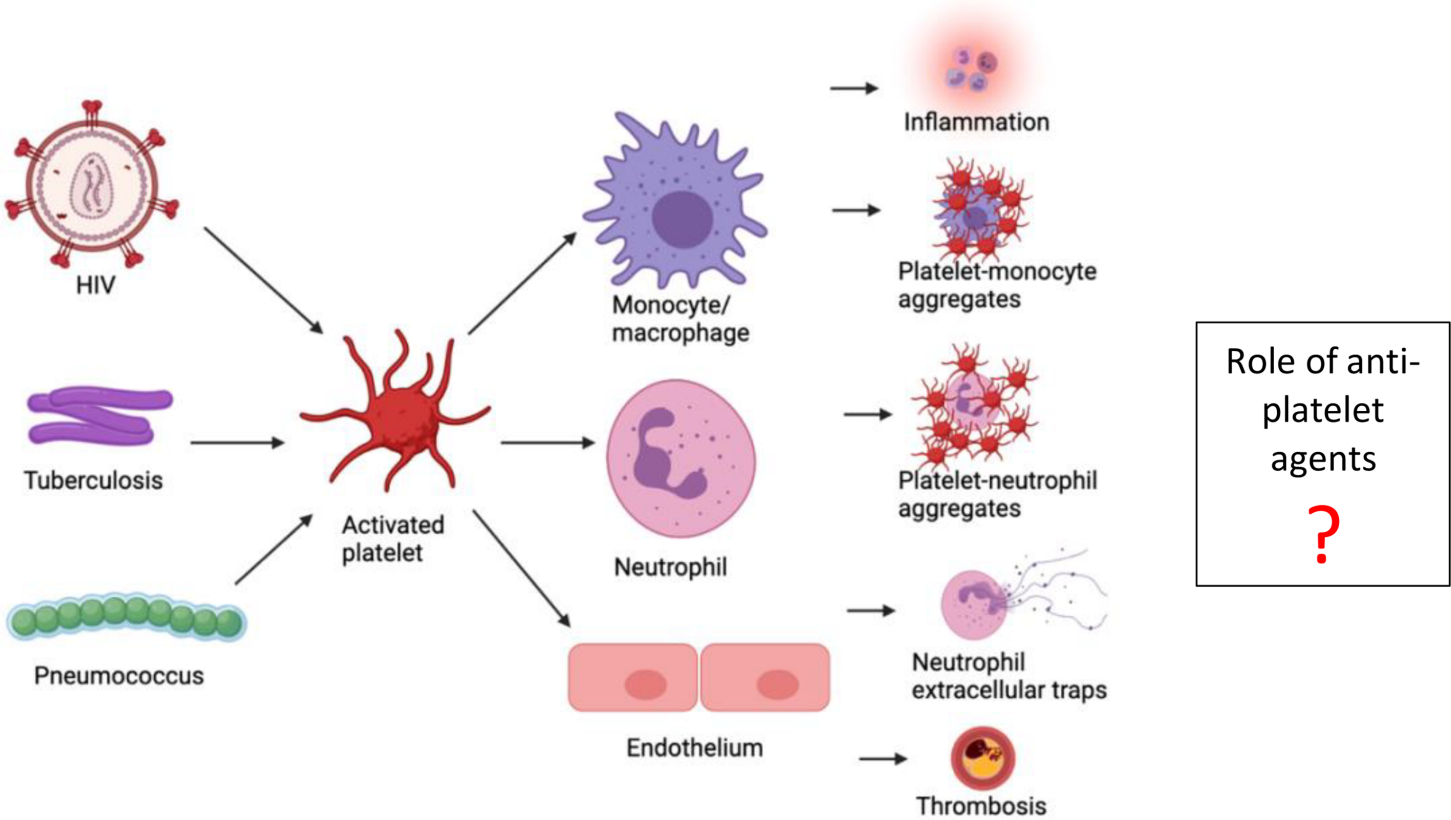
Summary of the role of platelet activation in the pathophysiology of HIV, tuberculosis and pneumococcal disease.

## Author Contributions

TR and CF contributed equally to this editorial. CF wrote the summary of the articles covering pneumococcus and tuberculosis and TR wrote the section about HIV and made the figure. Both authors contributed to the article and approved the submitted version.

## Conflict of Interest

The authors declare that the research was conducted in the absence of any commercial or financial relationships that could be construed as a potential conflict of interest.

## Publisher’s Note

All claims expressed in this article are solely those of the authors and do not necessarily represent those of their affiliated organizations, or those of the publisher, the editors and the reviewers. Any product that may be evaluated in this article, or claim that may be made by its manufacturer, is not guaranteed or endorsed by the publisher.
